# Roles of plant growth regulators on yield, grain qualities and antioxidant enzyme activities in super hybrid rice (*Oryza sativa* L.)

**DOI:** 10.1186/1939-8433-6-9

**Published:** 2013-04-16

**Authors:** Shenggang Pan, Fahd Rasul, Wu Li, Hua Tian, Zhaowen Mo, Meiyang Duan, Xiangru Tang

**Affiliations:** College of Agriculture, South China Agricultural University, Guangzhou City, Guangdong Province China; Scientific Observing and Experimental Station of Crop cultivation in South China, Ministry of Agriculture, Beijing City, China; Department of Agronomy, University of Agriculture Faisalabad, Faisalabad, Pakistan; Crops Research Institute, Guangdong Academy of Agricultural Sciences, Guangzhou City, Guangdong Province China

**Keywords:** Exogenous hormone, Protective enzyme, Rice (*Oryza sativa L*.)

## Abstract

**Background:**

Plant growth regulators play important roles in plant growth and development, but little is known about roles of plant growth regulators in yield, grain qualities and antioxidant enzyme activities in super hybrid rice. In this study, gibberellic acid(GA3), paclobutrazol (PBZ), 6-Benzylaminopurine(6-BA) treatments and distilled water (control) were sprayed to two hybrid rice cultivars (*Peizataifeng* and *Huayou 86*) at the heading stage in the field experiments in both early and late season in 2007. Treatments were arranged in a split-plot design with four replications. Cultivars treatments with two newly developed super hybrid rice *Peizataifeng* and *Huayou86* were the main plots and plant growth regulators treatments were the subplots. Subplot treatments included (1) plots sprayed with distilled water(CK), (2) plots sprayed with 20 mg L^-1^ GA3 prepared using 95% ethanol as surfactant(GA3), (3) plots sprayed with 50 mg L^-1^ PBZ(PBZ), (4) plots sprayed with 30 mg L^-1^ 6-BA(6-BA).

**Results:**

Spraying PBZ with 50 mg L^-1^ or 6-BA with 30 mg L^-1^ at the heading stage could increase the number of spikelets per panicle, seed setting rate and grain yields in *Peizataifeng* and *Huayou86* in both seasons. PBZ treatment also significantly improved head rice rate and amylose content in *Peizataifeng* and *Huayou86* in early season. Furthermore, it was observed that spraying PBZ or 6-BA could increase super oxide dismutase (SOD) and peroxidase (POD) activities, decrease accumulation of malendialdehyde (MDA) in flag leaves at the late growth stage.

**Conclusions:**

Application of PBZ or 6-BA partially alleviated the detrimental effects of rice senescence by modulating the activity of enzymatic antioxidants, and improving antioxidant system, which helped in sustaining plant growth. Therefore, spraying PBZ with 50 mg L^-1^ or 6-BA with 30 mg L^-1^ at the heading stage could increase grain yields and improve grain qualities in the two super hybrid rice.

**Electronic supplementary material:**

The online version of this article (doi:10.1186/1939-8433-6-9) contains supplementary material, which is available to authorized users.

## Background

Rice is the most important staple food in Asia, providing average 32% of total calorie uptake (Maclean et al., 2002). Mainly because of a still-growing population, demand for rice is expected to keep increasing in the coming decades (Pingali et al., 1997). At the current growth of population rice requirement increases dramatically and many nations are facing second-generation challenge of producing more rice at less cost in a deteriorating environment. Thus, improved technologies are required to achieve the goal of ensuring food security which is a challenging task. Therefore, enhancing productivity of rice through novel genetic approaches and exogenous plant growth regulators will be necessary.

Rice hybrids with a yield advantage of 20% were developed in China in the 1970s and are now planted in about 57% of the rice area in the country (Yuan, 2011). Plant growth regulators play vital roles in coordination of many growth and behavioral processes in rice, which regulates the amount, type and direction of plant growth (Rajendra and Jones Jonathan 2009; Anjum et al., 2011). The use of plant growth regulators, as GA3, PBZ, 6-BA or their compounds, is becoming popular to ensure efficient production. Remarkable accomplishments of plant growth regulators such as manipulating plant growth and crop yield have been actualized in recent years (Sarkar et al., 2002; Sakamoto et al.,^2005^; Morinaka et al., 2006; Yan et al., 2011; Zvi and Eduardo 2011). Plant growth regulators modify growth and development in various ways under different growth conditions. GA3 is responsible for stimulating the production of mRNA molecules in the cells, which in turn improves the chances of fast growth (Richards et al., 2001; Olszewki and Gubler 2002; Emongor, 2007). Nonstructural carbohydrates (NSC) and crude protein (CP) contents in rice straw were significantly increased by spraying GA3, especially on the 15th d after anthesis, and the fermentation quality of rice straw silage was improved with the increase of NSC and CP contents. Single panicle weight was also significantly increased by spraying GA3 after anthesis (Dong et al., 2012). Priming with GA3 was very effective in improving salinity-induced decrease in number of grains per ear on main stem in both wheat cultivars, which can alter the uptake and pattern of accumulation of different ions between shoots and roots in the adult plants of wheat under saline conditions. PBZ is a member of the triazole plant-growth inhibitor group. Like many plant growth regulators, triazoles have plant growth regulatory effects. Triazoles also increase tolerance of various plant species to biotic and abiotic stresses, including fungal pathogens, drought, air pollutants, and low and high temperature stress, by reducing oxidative damage via elevation of antioxidants or reducing the activity of oxidative enzymes (Lin et al., 2006; Baninasab Bahram, 2009). PBZ normally is applied as a foliar spray (Still and Pill, 2004). As one kind of cytokinin, 6-BA can reduce ethylene sensitivity of cut flowerers (Yuan et al., 2012). Exogenous 6-BA is able to inhibit the effects of ethylene, inhibit ethylene biosynthesis, induce 1-aminocyclopropane-1-carboxylate synthase (ACS) and 1-aminocyclopropane-1-carboxylate oxidase (ACO) gene expression and is involved in the early regulation of ethylene signal transduction in plants(Hall et al., 2001; Gapper et al., 2005). Zhang et al. (2007) reported that spraying external 6-BA on the leaves at late growth period of the late-season rice could increase seed setting rate and grain yield by delaying leaves senescence.

Two new super hybrid rice, *Peizataifeng* and *Huayou86,* were developed by College of Agriculture, South China Agricultural University (SCAU). *Peizataifeng* (Peiai64S × taifengzhan), which is a two-line hybrid rice. Its whole growth period is about 125 and 115 days for early and late planting in southern area of China, respectively. *Huayou86* is the new combination of late maturity-temperate three-line hybrid rice which has growth period of 130 days for early growing season and 115–120 days for late growing season in southern China, respectively. These two varieties have become important and popularized because of better growth characteristics in recent years (Jiang, 2008).

Published data have addressed effects of different plant growth regulators on rice growth and development (Yang et al., 2000; Bahram 2009; Yan et al., 2011). However, Southern China is one of the biggest double rice areas in China. *Peizataifeng* and *Huayou86* are the important popularized varieties in Southern China. Knowledge of plant growth regulation on these two newly developed rice hybrids is quite less in regional and international scientific literature. Therefore, the purpose of this study was to investigate the comparative effects of plant growth regulators (GA3, PBZ and 6-BA) on yield, grain quality and antioxidant enzyme activities in super hybrid rice*.*

## Results

### Grain yield and its components

Overall, foliar application of plant growth regulator at heading stage can increase grain yields in *Peizataifeng* and *Huayou86* in both early and late seasons in 2007(Table 1). In early season, grain yield of *Peizataifeng* under PBZ and 6-BA treatments was remarkably higher than that of CK, respectively. The higher yield was found for GA3, PBZ and 6-BA treatments, while the lowest one was found under control treatment. Significant differences were found in the number of spikelets per panicle and grain filling percentage between GA3, PBZ, 6-BA treatment and CK in two cultivars of *Peizataifeng* and *Huayou 86* in both early and late seasons in 2007. There was significant increase in the number of spikelets per panicle and grain filling percentage under plant growth regulator treatments in *Peizataifeng* and *Huayou86* compared with the CK, respectively.

**Table 1:** **Effects of plant growth regulators on yield and its components of cultivars of** ***Peizataifeng*** **and** ***Huayou 86*** **in both early and late seasons in 2007**

Treatments		Panicle number (m^-2^)	No. of Spikelets per panicle	Grain filling percentage (%)	1000-grain-weight (g)	Yield (kg ha^-1^)
2007 early rice						
*Peizataifeng*	CK	250 b	179.5 b	78.1 b	22.1 a	6807.7 b
	GA3	258 b	205.3 a	86.7 a	22.2 a	6565.0 b
	PBZ	258 b	198.0 a	85.8 a	23.1 a	7388.3 a
	6-BA	275 a	196.8 a	84.6 a	22.8 a	7626.7 a
	mean	260 A	194.9 A	83.8 A	22.5 A	7096.9 B
*Huayou86*	CK	228 b	140.6 a	80.4 b	19.8 b	7583.3 a
	GA3	271 a	122.9 b	86.2 a	20.0 b	7020.0 a
	PBZ	275 a	145.4 a	86.6 a	21.6 a	7930.0 a
	6-BA	258 ab	138.3 b	84.5 a	21.7 a	7940.7 a
	mean	258 A	136.8 B	84.4 A	20.7 B	7618.5 A
2007 late rice						
*Peizataifeng*	CK	273 a	135.0 b	80.8 b	21.7 b	7080.0 b
	GA3	276 a	141.4 ab	86.9 a	21.9 ab	7920.1 a
	PBZ	266 a	135.6 b	84.3 a	23.0 a	7740.0 ab
	6-BA	273 a	149.2 a	83.9 a	22.6 a	7680.7 ab
	mean	272 A	140.3 B	84.0 B	22.3 A	7605.0 B
*Huayou86*	CK	265 b	165.3 a	86.9 a	20.4 a	8280.0 a
	GA3	280 a	171.8 a	89.7 a	20.7 a	9040.0 a
	PBZ	269 b	168.3 a	88.9 a	20.4 a	8406.0 a
	6-BA	283 a	168.4 a	88.4 a	20.2 a	8620.0 a
	mean	274 A	168.5 A	88.4 A	20.5 B	8586.5 A

Within a column for two groups of genotypes, common letters are not significantly different at the 5% level.

Higher grain yield under GA3, PBZ and 6-BA treatments was mainly ascribed to the significant higher spikelets per panicle and grain filling percentage (Table 1). On average, grain yield of *Huayou 86* was higher than that of *Peizataifeng*. Average grain yield was higher in late season than that in early season.

### Grain qualities

There were significant differences in head rice rate, chalkiness rate and amylose content in the two cultivars between the treatments (Table 2). The PBZ treatment significantly increased head rice rate and amylose content in the cultivar *Peizataifeng* in early season. However, no remarkable increase was observed in late season in the cultivar Peizataifeng as compared to the CK. In late rice season, significant differences in amylose content were observed among these treatments (Table 2). No noticeable differences in brown rice rate and milled rice rate in the two cultivars were observed among the treatments in both early season and late season. Head rice rate under PBZ treatment was significantly higher than that the CK in early season, however, there were increases to some extent in head rice rate and amylose content in late season, but not significant as compared to the CK. Although PBZ treatment showed increase in the chalkiness of grain as compared to the CK, but the difference was not significant. PBZ treatment could improve grain’s milling quality trait and nutrition trait because of higher head rice rate and amylose content.

**Table 2:** **Effects of plant growth regulators on brown rice rate, milled rice rate, head rice rate, chalkiness and amylose content of cultivars** ***Peizataifeng*** **and** ***Huayou 86*** **in both early and late seasons in 2007**

Treatments		Brown rice (%)	Milled rice (%)	Head rice (%)	Chalkiness (%)	Amylose content (%)
2007 early rice						
*Peizataifeng*	CK	81.5 a	73.8 a	63.4 b	9.8 b	11.8 b
	GA3	81.6 a	74.3 a	66.2 ab	13.2 a	12.5 b
	PBZ	81.9 a	75.9 a	68.9 a	10.1 b	17.1 a
	6-BA	82.0 a	75.4 a	66.5 ab	13.6 a	17.6 a
	mean	81.7 A	74.9 A	66.3 B	11.7A	14.7 A
*Huayou86*	CK	81.4 a	75.4 a	66.0 b	12.9 a	15.5 a
	GA3	82.5 a	75.6 a	67.5 b	12.4 a	15.1 a
	PBZ	82.7 a	75.3 a	74.7 a	10.7 a	16.5 a
	6-BA	81.3 a	74.0 a	67.2 b	11.4 a	14.3 a
	mean	82.0 A	75.1 A	68.9 A	11.9 A	15.4 A
2007 late rice						
*Peizataifeng*	CK	82.0 a	76.3 a	67.7 a	8.3 ab	14.5 b
	GA3	82.4 a	77.2 a	70.0 a	10.1 a	17.9 a
	PBZ	82.1 a	76.8 a	69.7 a	9.2 a	17.2 a
	6-BA	82.0 a	77.4 a	69.7 a	6.7 b	17.2 a
	mean	82.1 A	76.9 B	69.3 A	8.6 A	16.7 A
*Huayou86*	CK	81.9 a	78.6 a	68.9 a	7.0 b	14.0 b
	GA3	81.7 a	78.6 a	70.1 a	8.7 b	18.5 a
	PBZ	82.2 a	79.0 a	71.3 a	12.1 a	18.7 a
	6-BA	81.2 a	79.0 a	70.1 a	10.1 ab	18.0 a
	mean	81.7 A	79.0 A	70.1 A	9.5 A	17.3 A

### Anti-oxidant enzyme activities

#### SOD activities

**Figure 1:**
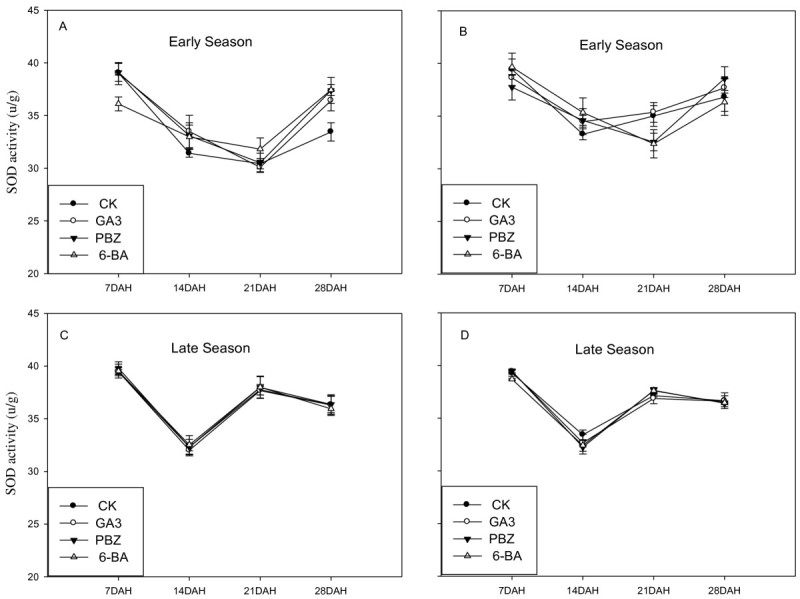
**Effects of plant growth regulators on SOD activity of rice flag leaves in the cultivars** ***Peizataifeng*** **and** ***Huayou86*** **in both early and late seasons in 2007. A** and **C**: *Peizataifeng*; **B** and **D**: *Huayou 86.*

Figure 1 showed the effects of different plant growth regulator treatments on SOD activity in rice leaves at different time intervals during early and late growing seasons of 2007. Both PBZ and 6-BA treatments increased significantly the activities of SOD in rice flag leaves at 14, 21 and 28 days after heading (DAH) for the cultivars *Peizataifeng* and *Huayou86* during early growing season, while no remarkable effect was observed for antioxidant activity at 14, 21 and 28DAH during the late growing season, compared with the CK. PBZ treatment showed the higher SOD activities during early growing season for cultivar *Peizataifeng* than *Huayou 86*. However, there was no significant difference in leaf SOD activity during late growing season. Generally, the SOD activities for the treatments of PBZ and 6-BA decreased slower with time after heading stage compared with the CK.

### POD activities

**Figure 2:**
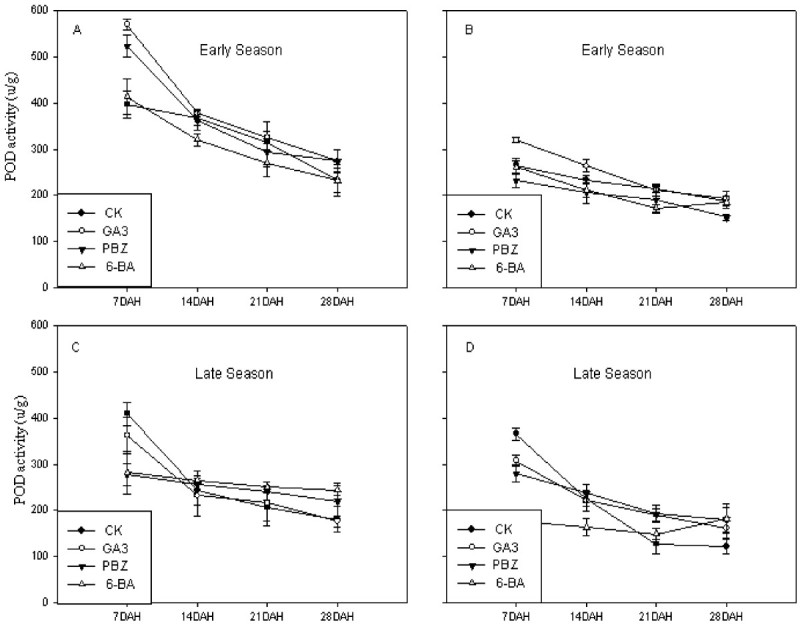
**Effects of plant growth regulators on POD activity of rice flag leaves in the cultivars** ***Peizataifeng*** **and** ***Huayou86*** **in both early and late seasons in 2007. A** and **C**: *Peizataifeng*; **B** and **D**: *Huayou 86.*

Figure 2 showed the effects of different plant growth regulators on POD contents in rice flag leaves at different time intervals during early and late growing seasons in 2007. Treatments of PBZ and 6-BA decreased significantly the activities of POD at 7, 14, 21 and 28DAH during early growing season compared to the CK (Figure 2). For example, leaf POD activities for the CK at 14, 21 DAH decreased from 362.30 Ug^-1^ to 292.90 Ug^-1^ for *Huayou 86* during early growing season, however, leaf POD activities for PBZ treatment at 14, 21DAH decreased from 233.33 Ug^-1^ to 188.47 Ug^-1^. The same trend happened under plant growth regulator treatment PBZ for *Peizataifeng* during early growing season. No remarkable differences were observed in leaf POD activities between GA3, PBZ, 6-BA treatment and CK in the two cultivars during late seasons in 2007. Overall, higher leaf POD contents were observed for *Peizataifeng* as compared to *Huayou86*, depicting a genotypic difference for leaf POD activity.

### MDA activities

**Figure 3:**
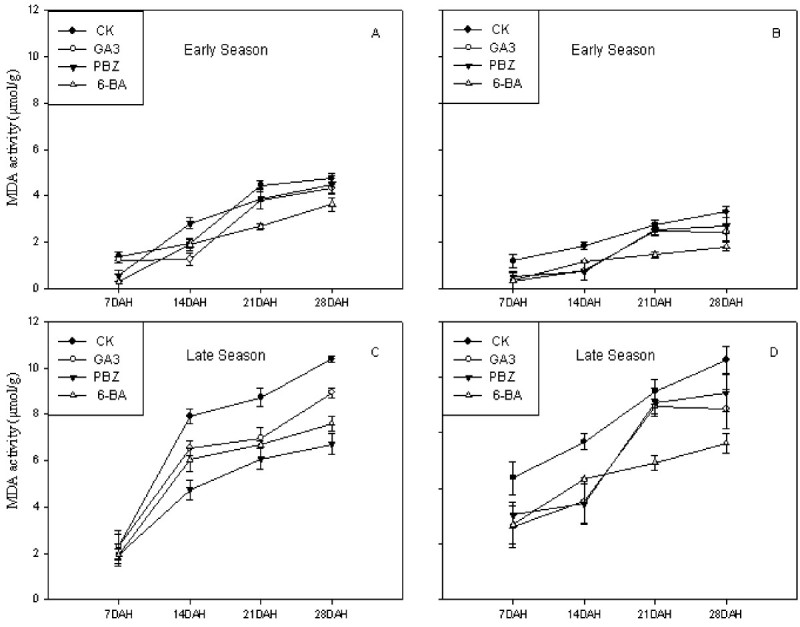
**Effects of plant growth regulators on MDA activity of rice flag leaves in the cultivars** ***Peizataifeng*** **and** ***Huayou86*** **in both early and late seasons in 2007. A** and **C**: *Peizataifeng*; **B** and **D**: *Huayou 86.*

Plant growth regulators decreased significantly the concentration of MDA of both cultivars at 7, 14 and 21DAH during both early and late growing seasons in 2007 (Figure 3). In early growing season, the concentration of MDA in flag leaves for the CK was from 1.40 μmol g^-1^ to 4.46 μmol g^-1^,while those for treatments PBZ and 6-BA decreased from 0.56 μmol g^-1^ to 3.88 μmol g^-1^, and 0.30 μmol g^-1^ to 2.68 μmol g^-1^ for cultivar *Peizataifeng* during early growing season, respectively. Similarly, the concentration of MDA in flag leaves for the CK was from 1.19 μmol g^-1^ to 2.75 μmol g^-1^, while those for treatments PBZ and 6-BA decreased from 0.52 μmol g^-1^ to 2.55 μmol g^-1^, and 0.35 μmol g^-1^ to 1.46 μmol g^-1^ for cultivar *Huayou86* during early growing season, respectively.

The same trend appeared for both cultivars *Peizataifeng* and *Huayou 86* during late growing season. MDA value in flag leaves of cultivar *Peizataifeng* for CK ranged from 2.31 μmol g^-1^ to 8.73 μmol g^-1^ at 7, 14 and 21DAH, while those for treatments PBZ and 6-BA decreased from 1.95 μmol g^-1^ to 6.07 μmol g^1^, and 1.93 μmol g^-1^ to 6.68 μmol g^-1^, respectively. The leaf MDA contents of all treatments started to increase progressively with the passage of time until 21 DAH after which MDA contents became almost stable. Overall, higher leaf MDA contents were observed for *Peizataifeng* than for *Huayou 86* depicting a genotypic difference for leaf MDA contents. Seasonal variation was also evident from the MDA contents values as average MDA contents were comparatively high at the late growing season when compared with the early growing season.

## Discussion

Plant growth regulators (GA3, PBZ and 6-BA) play important roles in plant growth, development, yield and qualities formation (Ekamber and Kumar 2007; Rajendra and Jones Jonathan 2009). Zheng et al. (2011) found that suitable application of plant growth regulators (such as NAA-Na,GA3 or 6-BA) could improve the photosynthetic capacity, delay the leaf senescence and promote the rate of rice seed-setting.In this study, we observed that spraying PBZ with 50 mg L^-1^ or 6-BA with 30 mg L^-1^ at the heading stage could also increase number of spikelets per panicle, seed setting rate and grain yields in both cultivars of *Peizataifeng* and *Huayou 86* in both seasons. There was more noticeable effect on yield in *Huayou 86* than in *Peizataifeng* with foliar application of PBZ or 6-BA. This result agrees with the findings of Peng et al. (2011), who found that spraying PBZ in rice had more effective grain number, seed setting rate, 1000-grain weight, and yield was increased by 11.89% under PBZ treatment compared with the CK. Furthermore, the application could enhance the lodging resistance and obviously increase root biomass and root activity to improve phosphorus and potassium accumulation in rice stem, leaves and grains. SOD and POD are important antioxidant enzymes that detoxify active oxygen species. Antioxidant enzymes aid cells in removing harmful oxygen species. We also found that spraying PBZ or 6-BA could increase SOD and POD activities, and decrease accumulation of MDA in flag leaves at the late growth stage. This result suggested that PBZ or 6-BA application partially alleviated the detrimental effects of rice senescence by modulating the activity of enzymatic antioxidants, improving antioxidant system, which helped in sustaining plant growth and yield.

Grain quality is one of the most important traits in evaluating planting rice’s benefit. There were significant effects on grain qualities by spraying exogenous plant growth regulator. Du et al. (2010) studied that the inferior grains plumpness in rice was enhanced 9.7% and 5.5% by spraying exogenous 6-BA and GA3 at 5 days before flowering, respectively. Application of 6-BA decreased the chalky rice rate and chalky area of grain and chalk degree compared to the CK, respectively. Furthermore, exogenous hormones had greater effects on grain qualities of inferior spikelets than superior spikelets. Dong et al. (2009) reported that the effects of exogenous hormones on rice quality varied with exogenous hormone varieties and different grain positions by spraying GA3 (57.7 μmol ^-L^) at earlier filling stage in rice. The exogenous GA3 decreased 1000-grain-weight and head rice rate and protein content, but increased chalkiness and amylose content. In present study, the effect of spraying GA3 on 1000-grain-weight and head rice rate of the earlier-flowered spikelets was greater than that of the later-flowered spikelets. PBZ treatment significantly increased head rice rate and amylose content in the cultivars *Peizataifeng* and *Huayou86* in early season, however, the chalkiness of grains was not increased significantly. Our results did not agree with previous studies fully (Dong et al., 2009). Variations may have arose due to experimental materials, plant growth regulator concentration and application time. It is, therefore, possible that foliar plant growth regulator could be a useful tool in promoting grain yield and bettering quality in rice. Whether or not this would be sufficient to promote adequate rice production for the whole cycle would require further more detailed and longer-term investigation in the aspects of a diverse group of rice genotypes and physiological and biochemical mechanism arising from plant growth regulator’s influence.

## Conclusions

Spraying PBZ with 50 mg L^-1^ or 6-BA with 30 mg L^-1^ at the heading stage could increase spikelets per panicle, seed setting rate and grain yields in the cultivars *Peizataifeng* and *Huayou 86* in both seasons. PBZ treatment also significantly improved head rice rate and amylose content in two cultivars of *Peizataifeng* and *Huayou 86* in early season. Furthermore, it was observed that spraying PBZ or 6-BA could increase SOD and POD activities, decrease accumulation of MDA in flag leaves at the late growth stage. Application of PBZ or 6-BA partially alleviated the detrimental effects of rice senescence by modulating the activity of enzymatic antioxidants, improving antioxidant system, which helped in sustaining plant growth.

## Methods

### Field experiments

Field experiments were conducted in the early season (March-July) and repeated in the late season (July to November) in 2007, at the College of Agriculture’s Experimental Farm, South China Agricultural University (SCAU), Guangzhou, Guangdong province, China (113.18′E, 23.10′N, elevation 18 m). The properties of soil collected from the upper 20 cm are as follows, 22.0 g kg^-1^ organic C, available N 105.7 mg kg^-1^, available P 128.3 mg kg^-1^(water as extract), available K 112.5 mg kg^-1^(ammonium acetate(1 mol l^-1^) as extract).

Treatments were arranged in a split-plot design with four replications. Main plots were varieties with two newly developed super hybrid rice *Peizataifeng* and *Huayou86*. *Peizataifeng* was developed by College of Agriculture, South China Agricultural University (SCAU), (*Peiai64S* × *taifengzhan*), which is a two-line hybrid rice. Its whole growth period is about 125 and 115 days for early and late planting, respectively. *Huayou86* is new treatment combination of late maturity-temperate three-line hybrid rice. Whole growth period in Guangdong province is 130 days for early growing season and 115–120 days for late growing season, respectively. Subplot treatments included (1) the CK, (2) GA3 treatment, (3) PBZ treatment, and (4) 6-BA treatment. All plant growth regulators above were purchased from Xiamen Top using Chemical Co., Ltd (Fujian, P.R. China).

Twenty-day-old seedlings from wet bed nurseries were transplanted at the rate of 2 seedlings per hill at a spacing of 20.0 cm × 20.0 cm (2.5×10^5^ hills ha^-1^) on 5^th^ of April and 12^th^ of August, respectively. Phosphorus in the form of single superphophate (SSP) was applied at the rate of 67.5 kg P_2_O_5_ ha^-1^ as basal dose. Potassium (potassium chloride) at 162 kg K_2_O ha^-1^ was applied with a split of 60% as basal dose and 40% at panicle initiation stage (PI). Nitrogen (urea) was applied with 50% as basal dose, 30% at mid-tillering (MT) and 20% at PI. The area of each sub-plot was 10 m^2^. The field was kept flooding from transplanting until 10 days before maturity when the field was drained. Disease and weeds were intensively controlled to avoid yield loss.

### Foliar application of growth regulators

The solution of GA3 (20 mg L^-1^), PBZ (50 mg L^-1^), and 6-BA (30 mg L^-1^) was prepared, respectively. Foliar application of growth regulators was carried out at the heading stage by spraying plants uniformly to the point of run-off (approximately 100 mL m^-2^) using a Gloria type hand sprinkler (Guangzhou, P.R. China) with constant flow. Each plot was sprayed with 1.0 L of the specific solution. Control plot was treated with water. The treatments were applied late in the afternoon. The concentration of each substance was selected on the basis of previous experiments conducted since 2005 by our laboratory to establish optimum dosages for various rice cultivars, including the the two cultivars used in this study, and on actual practices employed by farmers in Guangzhou.

### Plant samplings and enzymes activities measurement

Twenty flag (Top) leaves from each treatment were sampled at random and treated with liquid nitrogen for 1 min. Then they were stored at −80°C for enzymes activities measurements i.e. SOD, POD and MDA at 7, 14, 21 and 28 days after heading. The level of leaf senescence was determined by measuring the amount of MDA, which is the end product of lipid peroxidation, following the method of De Vos et al. (1991). Leaf samples (0.5 g) were homogenized in 5 mL of 5% trichloroacetic acid. The homogenate was centrifuged at 4 000× g for 10 min at 25°C and 3 mL of 2-thiobarbituric acid in 20% trichloroacetic acid was added to a 2 mL aliquot of the supernatant. The mixture was heated at 98°C for 10 min and cooled rapidly in an ice bath. After centrifugation at 4 000× g for 10 min, the absorbance was recorded at 532 nm. Measurements were corrected for non-specific turbidity by subtracting the absorbance at 600 nm. Concentration of MDA was determined by extinction coefficient MDA (ε= 155 μm cm^-1^).

SOD was measured according to the method described by Beauchamp and Fridovich (1971) using nitro blue tetrazolium (NBT). The assay mixture comprised of 1.5 mL of 0.1 mol L^-1^ phosphate buffer(PB, pH=7.5), 0.3 mL of 1.3 mol L^-1^ Methionine, 0.3 mol of 750 umol L^-1^ NBT, 0.3 mL of 100 umol L^-1^ EDTA-Na_2_, 0.3 mL of Riboflavin and 0.25 mL of distilled water. A total volume of 3.0 mL of assay mixture was reached by adding 0.05 mL of enzyme extract (0.05 mL of phosphate buffer saline (PBS) for the blank test). The assay mixture was exposed later uniformly under a light source of 4000×g for 20 min. Under these conditions riboflavin was excited by a photon and was able to oxidize an electron donor molecule in this case methionine. This donation of an electron results in the production of a superoxide molecule (O_2_^-^). The O_2_^-^ molecule was able to reduce the NBT, giving an insoluble purple formazan. This colour change can be measured by spectrophotometer at A560 nm. The presence of SOD leads to a reduction in the level of formazan being produced. One unit of SOD was defined as the amount of enzyme necessary to produce a 50% inhibition of the maximum value of inhibition. It should be noted that even extremely concentrated forms of the enzyme never lead to a 100% inhibition, and so the 50% mark was defined as being the midpoint between no inhibition and the point where maximal (but not complete) inhibition takes place.

The peroxidase (POD) activity was determined using the method of Cai et al. (2008). Fresh leaf segments(<2 mm, 0.25 g) were homogenized in an ice bath in 5 mL of 50 mM borate buffer(pH 8.7) containing 5.0 mM sodium hydrogen sulfite and 0.1 g polyvinylpyrrolidone (PVP) The homogenate was centrifuged at 9000 g for 15 min at 4°C. The supernatant was used as enzyme extract. POD activity was assayed by adding 0.1 mL of the enzyme extract to a substrate mixture containing acetate buffer (0.1 mol L^-1^, pH 5.4), ortho-dianisidine (0.25% in ethyl alcohol) and 0.1 mL 0.8% H_2_O_2_ was added to 0.1 mL of the enzyme extract. Absorbance change of the brown guaiacol at 460 nm was recorded for calculating POD activity. One POD unit of enzyme activity was defined as the absorbance increase because of guaiacol oxidation by 1 unit min^-1^ (U g^-1^ FW min^-1^).

### Harvesting and grain quality measurements

Yield and its components were determined according to the method described by Peng et al. (2006) with minor modification. At maturity, 30 hills of plants from each sub-plot were investigated for calculation of the average valid panicle numbers per hill. Then, six representative hills of the plants were separately sampled and investigated for yield components. Panicles were hand-threshed and ripened grains were separated from unripe grains by submerging them in tap water. Three subsamples of 30 g of ripened grains and 5 g of unripe grains were taken to count the number of spikelets. The ripened grains were then oven-dried at 70°C until constant weight for determining grain weight. Percentage of ripened grains (100 × ripened grains number/total grain number), and harvest index (100 × ripened grains weight /aboveground total biomass) were calculated. Grain yield was determined by harvesting an area of 5 m^2^ in the centre of each sub-plot (excluding the border lines) and adjusted to the standard moisture content of 0.14 g H_2_O g^-1^.

Grain quality measurements were determined according to the method described by Zhang et al. (2008). About 500 g of grains harvested from each subplot were dried at 40°C in a forced-air oven for quality analysis. A 150 g sample of rice grains were twice passed through a dehusker, polished, then separated into broken and unbroken grains. The brown rice rate, milled rice rate, and head rice rate were expressed as percentages of total (150 g) rice grains. Chalkiness was evaluated visually on 100 milled grains per subplot. Grains containing 20% or more white belly, white centre, and white back or a combination of these were considered chalky. Amylose content and protein content were measured according to Rice Quality Measurements Standards (Ministry of Agriculture, PR China, 1988).

### Statistical analysis

Analyses of variances (ANOVA) were made with the General Linear Model Procedure of SAS statistical software version 8.1 (SAS Institute 2003). ANOVA was used to determine the treatment effects on each variable and confidence intervals derived from these analysis used to establish significant effects. Tukey’s test was used for comparison of significant effects.

## Authors’ original submitted files for images

Below are the links to the authors’ original submitted files for images. Authors’ original file for figure 1 Authors’ original file for figure 2 Authors’ original file for figure 3

## Abbreviations

DAH: Days after heading
GA3: Gibberellic acid
MDA: Malendialdehyde
PBZ: Paclobutrazol
POD: Peroxidase
SOD: Super oxide dismutase
6-BA: 6-Benzylaminopurine.
