# Supplementary figures and images for: Roles of plant growth regulators on yield, grain qualities and antioxidant enzyme activities in super hybrid rice (Oryza sativa L.)
Source: Rice (N Y). 2013 Apr 16;6:9. doi: 10.1186/1939-8433-6-9 (PMC4883720; doi:10.1186/1939-8433-6-9)

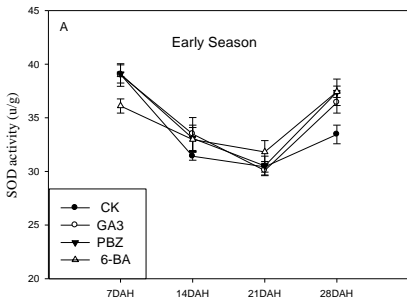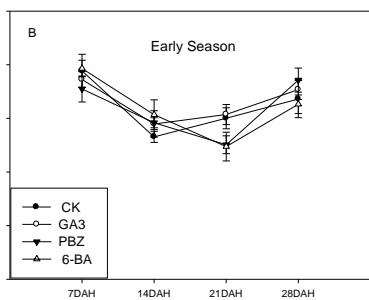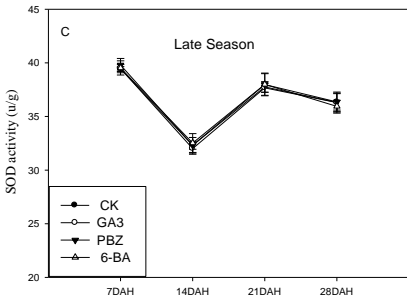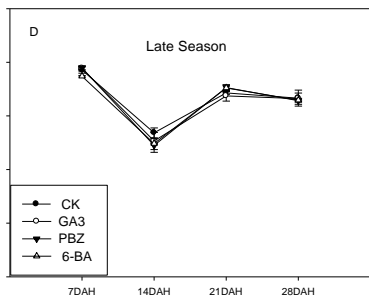

Supplement: Supplementary file 1 — Authors’ original file for figure 1 [file 12284_2012_47_MOESM1_ESM.pdf]

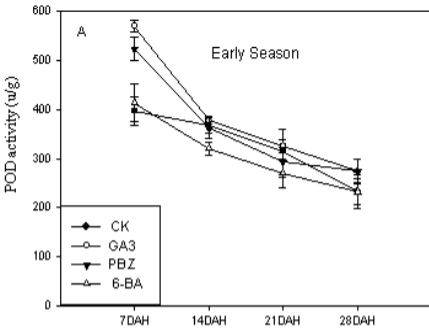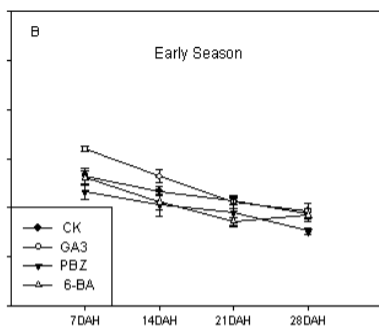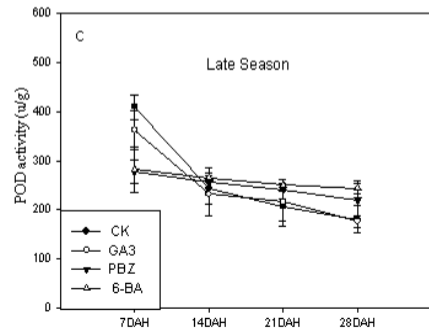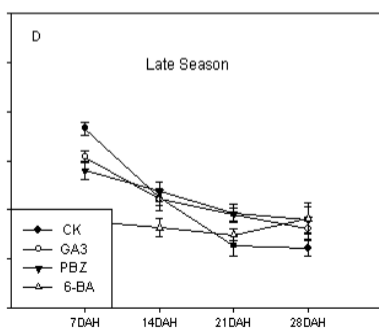

Supplement: Supplementary file 2 — Authors’ original file for figure 2 [file 12284_2012_47_MOESM2_ESM.pdf]

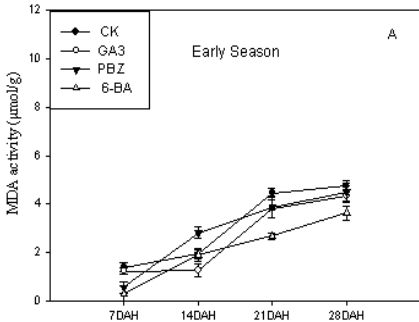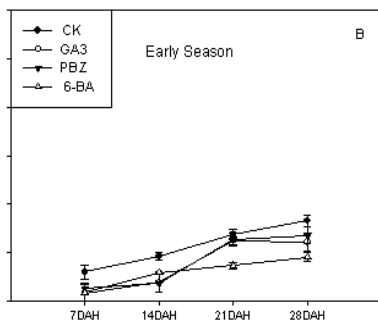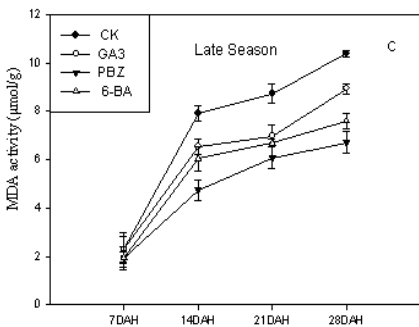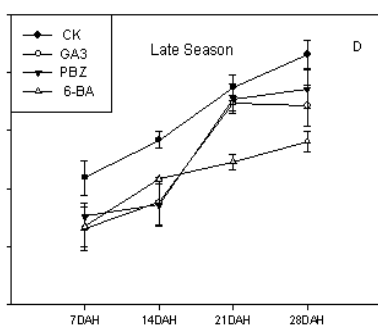

Supplement: Supplementary file 3 — Authors’ original file for figure 3 [file 12284_2012_47_MOESM3_ESM.pdf]
